# FLOT Neoadjuvant Chemotherapy Followed by Laparoscopic D2 Gastrectomy in the Treatment of Locally Resectable Advanced Gastric Cancer

**DOI:** 10.1155/2020/1702823

**Published:** 2020-05-29

**Authors:** Shun Zhang, Dongyi Yan, Qi Sun, Tao Du, Dongliang Cao, Yao Yang, Biao Yuan, Haiqiang Li, Xiaohua Jiang, Chun Song

**Affiliations:** ^1^Department of Gastrointestinal Surgery, Shanghai East Hospital (East Hospital Affiliated to Tongji University), Shanghai, China; ^2^Department of Colorectal Surgery, Jiangyin People's Hospital, Jiangyin, China

## Abstract

**Background:**

The prognosis of patients with advanced gastric cancer remains unsatisfactory, highlighting the need for improved therapeutic strategies. We analyzed 23 resectable advanced gastric cancer patients who received FLOT followed by laparoscopic gastrectomy with D2 lymphadenectomy to evaluate the efficacy and safety.

**Methods:**

Patients aged 18–75 years with gastric adenocarcinoma (stage cT3–4 and/or N + M0) underwent neoadjuvant FLOT therapy (four preoperative and four postoperative 2-week cycles) at Shanghai East Hospital. Laparoscopic gastrectomy was scheduled 3-4 weeks after completion of the last cycle of preoperative chemotherapy. The type of surgical procedure was determined by the location and extent of the primary tumor.

**Results:**

23 patients were reviewed in the study. 20 patients (81.2%) received four courses of FOLT therapy, while 3 patients (18.8%) received three courses of treatment. There were 3 (13.0%) complete responses, 13 (56.5%) partial responses, 4 (26.1%) of stable disease, and 1 (4.3%) of progressive disease. The clinical efficacy response rate was 69.6%. The R0 resection rate was 91.3%. Only one patient exhibited grade III postoperative complications. The pathologic complete remission was 13%. The common grade 3/4 adverse events from chemotherapy were leucopenia (17.4%), neutropenia (30.4%), anemia (13%), anorexia (13%), and nausea (17.4%). Postoperative complications occurred in 5 patients (26.1%). There was no treatment-related mortality or reoperation. The most reason for not completing chemotherapy was the patient's request.

**Conclusions:**

These findings suggest that FLOT neoadjuvant chemotherapy, followed by laparoscopic D2 gastrectomy, is effective and safe in advanced, resectable advanced gastric cancer.

## 1. Introduction

Gastric cancer is one of the most common malignancies in Asia, especially in China [1], even though its overall incidence has been decreasing considerably for the last 20 years. Surgery alone often does not provide a cure, especially in the advanced stage. Two landmark clinical trials of perioperative chemotherapy, the MAGIC trial [2] and French FNCLCC/FFCD 9703 [3], showed increased overall survival at five years compared with surgery alone.

The purposes of neoadjuvant chemotherapy are not only to evaluate the susceptibility and tolerability to chemotherapeutic agents but also to reduce the local tumor recurrence, improve overall survival (OS) by downstaging the tumor, increase pathology response, and allow for subsequent R0 resection [4].

However, the prognosis for patients with locally advanced gastric cancer remains unsatisfactory, highlighting the requirement of novel therapeutic strategies. It was reported that the docetaxel-based triplet FLOT (fluorouracil plus leucovorin, oxaliplatin, and docetaxel) protocol increased rates of curative surgery and prolonged OS and progression-free survival (PFS) as compared to ECF/ECX (epirubicin, cisplatin, fluorouracil/capecitabine) [5]. Recently, the overall survival results of the expanded population of the FLOT phase 3 trial were released. Median overall survival increased by 15 months and the estimated 2-year, 3-year, and 5-year survival rates by 9%, and the benefit in survival is clinically meaningful [6].

After neoadjuvant chemotherapy, the followed gastrectomy is another important factor to determine the treatment effect. Laparoscopic gastrectomy has been accepted to treat early-stage gastric cancer. Locally advanced gastric cancer is more challenging. A few randomized controlled trials (RCTs) and retrospective studies were performed to compare laparoscopic with open gastrectomy in locally advanced gastric cancer. Recent results of the open-label CLASS-01 trial showed that laparoscopic gastrectomy did not lead to inferior 3-year disease-free survival (DFS) [7]. But in this clinical trial, none received neoadjuvant chemotherapy.

To our knowledge, there were very few studies about neoadjuvant chemotherapy followed by laparoscopic gastrectomy, and there was no study on FLOT neoadjuvant chemotherapy followed by laparoscopic gastrectomy. There were also few studies showing the result of FLOT neoadjuvant chemotherapy applied in Asian patients. We report 23 resectable locally advanced gastric cancer patients who received FLOT neoadjuvant chemotherapy followed by laparoscopic gastrectomy with D2 lymphadenectomy. The aim of the current study is to clarify the oncology outcomes of FLOT neoadjuvant chemotherapy, especially in Chinese patients. We also want to study the role of laparoscopic gastrectomy following neoadjuvant chemotherapy. We found that patients who received FLOT combined with laparoscopic D2 gastrectomy got satisfactory results in clinical efficacy, adverse effects, postoperative complications, and pathological response.

## 2. Method

### 2.1. Patients

Patients with locally advanced gastric cancer who had received FLOT neoadjuvant chemotherapy followed by laparoscopic gastrectomy in the Gastrointestinal Department of the Shanghai East Hospital Tongji University between December 2016 and April 2018 were reviewed. All patients were diagnosed as histologically proven and clinically resectable advanced gastric cancer through enhanced computed tomography (CT), endoscopic ultrasound (EUS), and biopsy. Multidetector spiral CT (Brilliance 64, Philips Medical Systems, Cleveland, OH, USA) is used for enhanced CT scanning. Patients need to fast for 12 h before CT. In order to reduce gastrointestinal peristalsis, anisodamine was intramuscularly injected 10 to 20 min before the examination. In order to expand the stomach cavity, patients should take 500 to 700 mL of degassed water 10 min before the examination. A plain scan with dynamic contrast imaging was conducted with a 3 mm slice thickness, using Ultravist (Iopromide 300; Bracco Sine, Shanghai, China) as the contrast agent. EUS was performed using the Olympus processor with a standard radial transducer (Olympus). The gastric lumen was filled with 300 to 600 mL of degassed water to improve the transmission of the ultrasound beam with variable frequencies. The tumor infiltration depth was imaged as a hypoechoic disruption and evaluated based on the 5-layered gastric wall structure. The TNM categories were based on the Union for International Cancer Control tumor-node-metastasis classification [8]. All patients were informed about the adverse effects accompanying therapies and they all signed informed consent forms.

### 2.2. Inclusion Criteria

1. Preliminary pathologically confirmed gastric cancer based on an endoscopic biopsy
2. No prior antitumor therapy
3. Locally advanced gastric cancer (stage cT3–4 and N + M0) according to EUS and CT examinations
4. Good performance status (0-1) of Eastern Clinical Oncology Group (ECOG), age from 18 to 75 years, normal hematopoietic, hepatic and renal functions, and peripheral neuropathy grade < 2
5. Written informed consent for neoadjuvant chemotherapy before surgery

### 2.3. Neoadjuvant Chemotherapy

Patients were administered four cycles of neoadjuvant chemotherapy before surgery. The FLOT chemotherapeutic treatment was as follows: intravenous docetaxel, 50 mg/m^2^; intravenous oxaliplatin, 85 mg/m^2^; intravenous leucovorin, 200 mg/m^2^; and fluorouracil, 2600 mg/m^2^ as a 24 h infusion, all on day 1. All drugs were administered in a cycle of 14 days.

### 2.4. Clinical Efficacy Assessment

After four cycles of FLOT, gastroscopy, enhanced CT, or MRI was used to assess the tumor response to neoadjuvant chemotherapy by calculating the tumor size and lymph node metastasis. The clinical efficacy response was evaluated using the response evaluation criteria in solid tumors (RECIST 1.1) [9] guidelines. Response criteria include complete remission (CR), partial remission (PR), stable disease (SD), and progressive disease (PD).

### 2.5. Toxicity

Toxicity of neoadjuvant chemotherapy was graded (0-IV) to the Common Terminology Criteria for Adverse Events (CTCAE), version 4.0.

### 2.6. Laparoscopic Gastrectomy and Pathological Response

Laparoscopic gastrectomy was scheduled at least 4 weeks after the end of the fourth cycle of neoadjuvant chemotherapy. The type of gastrectomy was based on the location and extent of the primary tumor. D2 lymph node dissection was performed according to the guidelines of the Japanese Gastric Cancer Association (JGCA) [10]. Laparoscopic gastrectomy was performed using 5 trocars. First, routine exploration of the tumor site and the peritoneal cavity was performed to determine whether the tumor was transmural and to exclude peritoneal metastasis before resection. After radical lymphadenectomy, a minilaparotomy was performed for specimen extraction and anastomosis. The specimen was removed and grossly checked. Frozen section diagnosis on proximal or distal resection margins was performed routinely in all patients. Gastrointestinal tract continuity was performed in Billroth I or Roux-en-Y reconstructive procedures in distal gastrectomy and in Roux-en-Y procedure in total gastrectomy.

All specimens were locally reviewed by an experienced histopathologist. The pathological response assessment was scored using the tumor regression grade (TRG) of the Becker criteria [11]. The Becker criteria were graded as follows: TRG1a (no residual tumor), TRG1b (<10% residual tumor per tumor area), TRG2 (10%–50%residual tumor per tumor area), and TRG3 (>50% residual tumor per tumor area). Patients with the lack of all signs of cancer in the surgical specimens and retrieved lymph nodes were defined as pathologic complete remission (pCR). Radical resection (R0) was determined as a microscopically margin-negative resection, in which no macroscopical or microscopical tumor leaves behind. R1 resection indicates the removal of all macroscopic disease, but microscopic margins are positive for tumor.

### 2.7. Postoperative Complications

Postoperative complications were defined as any deviation occurred within 30 days after surgery. Postoperative complications were stratified using the Clavien–Dindo classification [12].

### 2.8. Statistical Methods

Descriptive statistics were calculated for patients' characteristics using mean, standard deviation, and percentages. All statistical data were performed using the SPSS 16.0 statistics software.

## 3. Results

### 3.1. Patients' Characteristics

Twenty-three patients were enrolled between December 2016 and April 2018.  Table 1 showed patient characteristics, histological subtype, and preoperative TNM stages. Most of the patients were male and over 80% had an ECOG PS of 0. The most frequent tumors were poorly differentiated adenocarcinoma. Twenty patients (81.2%) received four courses of FLOT therapy, while 3 patients (18.8%) received three courses of treatment.

### 3.2. Clinical Efficacy Assessment to Neoadjuvant Chemotherapy

Three (13.0%) patients observed CR after neoadjuvant chemotherapy. Thirteen (56.5%) patients showed PR, while SD and PD were observed in 6 (26.1%) and 1 (4.3%) patients, respectively. Among the 23 patients, the clinical efficacy response rate was 69.6% (16/23).

### 3.3. Adverse Effects in the FLOT Group

The toxicities through the neoadjuvant chemotherapy are listed in Table 2. The most common adverse events of grade 3/4 from chemotherapy were neutropenia (30.4%), leucopenia (17.4%), nausea (17.4%), anemia (13%), anorexia (13%), and vomiting (8.7%). No patients needed treatment termination and also no death occurred as a result of toxicities.

### 3.4. Operative Outcomes

All 23 patients received laparoscopic D2 radical gastrectomy after 3 to 4 weeks of the last cycle of neoadjuvant chemotherapy. 12 patients underwent total gastrectomy and 11 patients received distal gastrectomy. Surgical outcomes are shown in Table 3. The R0 resection rate was 91.3% (21/23). The median operation time was 225 minutes and the mean estimated blood loss was 105 mL. Median hospitalization duration was 12 days (range, 9–35).

### 3.5. Postoperative Complications

The incidence of postoperative morbidities was as follows: comprising surgical site infection in 3 patients, pneumonia in 1 patient, pancreatic fistula in 1 patient, and intra-abdominal abscess in 1 patient. There was no operative related mortality and no patients needed reoperation. The results of postoperative complications according to Clavien–Dindo classification are shown in Table 4. Only one patient exhibited grade III complications. One patient with pancreatic fistula was treated with conservative treatment and had the longest recovery time (31 days). The complications of pneumonia and intra-abdominal abscess prolonged the time of the postoperative hospital stays (20 days and 25 days, respectively).

### 3.6. Pathological Response

The pathological response of neoadjuvant chemotherapy was TRG1a in 3 (13.0%) patients, TRG1b in 6 (26.1%) patients, TRG2 in 8 (34.8%) patients, and TRG3 in 6 (26.1%) patients. In the study, pCR rate was 13.0%.  Table 5 shows the pathological findings in all resected patients.

### 3.7. Postoperative Treatment

Postoperative four-cycle adjuvant chemotherapy using FLOT was administered to 18 of 23 patients who underwent curative gastrectomy. One patient received only S-1 adjuvant chemotherapy due to patient refusal of intravenous chemotherapy. Four patients refused to receive any adjuvant chemotherapy.

### 3.8. Follow-Up

The last follow-up was in April 2019, the follow-up period ranged from 6 to 24 months, and the median follow-up was 12 months. At the end of the follow-up period, 19 patients survived, 3 patients died, and 1 patient was lost to follow-up. Recurrences were detected in 5 patients, of which 1 was lung metastasis, 2 were liver metastases, and 2 were multiple-organ metastases.

## 4. Discussion

Despite its declining incidence, gastric cancer is still the third leading cause of cancer deaths worldwide. China has the highest incidence of gastric cancer, accounting for more than 40% of the annual cancer incidence in the world [1]. Due to lacking specific symptoms in the early stage, advanced gastric cancer is more common with more than 80% among all diagnosed patients in China [13]. With the development of instruments and accumulation of experience, the surgeon can assess preoperative staging more accurately using new diagnosis technologies such as EUS and enhanced CT [14–16]. Although improvements in adequate lymph node dissection, the majority of advanced gastric cancer patients treated with surgery alone cannot successfully undergo R0 resection and relapse with high recurrence and mortality rates. Now, there is a standard consensus worldwide regarding combination neoadjuvant chemotherapy followed by surgery in resectable GC patients, as a means of resulting in tumor downstaging, improving resectability, and eradicating micrometastases, particularly in patients diagnosed at an advanced stage [17].

Two pioneering perioperative chemotherapy clinical trials indicated a significant increase in DFS and OS rate in advanced gastroesophageal junction or GC. The MAGIC trial was the first one to show a survival benefit of surgery combination with perioperative chemotherapy in advanced gastric cancer [2]. The results showed that the perioperative regimen of ECF (epirubicin, cisplatin, and infused fluorouracil) significantly decreased tumor size and induced downstaging and a significant prolongation of 5-year overall survival rate from 23% in the surgery alone group to 36% in the surgery combined with perioperative chemotherapy group. The French ACCORD07/FFCD 9703 multicenter phase-III trial showed that preoperative chemotherapy with infused fluorouracil-cisplatin can significantly improve DFS and OS [3].

However, the management of adjuvant chemotherapy in clinical practice is usually more complex due to toxicity, effectiveness, and cost performance in China. There is a need for a more effective neoadjuvant treatment. Combination chemotherapy therapies have been associated with substantially higher response rates and improved survival benefit compared to monotherapy. There is currently no consensus regarding whether triplet or doublet chemotherapy should be used as a first-line treatment for advanced gastric cancer patients. Naj Mohammad et al. conducted a metastudy to show whether triplet chemotherapy had more efficacy and safety of triplet than doublet chemotherapy in patients with locally advanced or metastatic esophagogastric carcinoma. They found that patients treated with triplet protocols (taxane, cisplatin, and fluoropyrimidine) can get a more significant benefit in the subgroup analysis [14, 18]. Among the triplet protocols, there was also a debate about which protocol had more safety and effectiveness. Epirubicin-containing chemotherapy protocol (epirubicin, cisplatin/oxaliplatin, and capecitabine (ECX/EOX)) is commonly used in the preoperative treatment, as recommended by ESMO Clinical Practice [15, 19]. But several studies showed that docetaxel-containing chemotherapy protocol was more effective as preoperative treatment. Yoon el al. conducted a phase II clinical study about docetaxel, capecitabine, and cisplatin (DXP) triplet chemotherapy in advanced gastric cancer. The result showed that DXP was safe and might be better in terms of recurrence-free survival (RFS) [16, 20]. Swiss group for clinical cancer research conducted a randomized phase II clinical trial to make a comparison between docetaxel + cisplatin + 5FU (DCF) and ECF to see which would be most promising according to the overall response rate (ORR) [17, 21]. The result showed that the protocol containing DCF seemed to be more effective than ECF. DCF had a shorter time to respond, which may indicate that docetaxel-containing protocols are more suitable as neoadjuvant treatment compared to epirubicin-containing chemotherapy protocols. Recently, the analysis results of the multicenter, randomized, phase 3 FLOT4-AIO trial have been presented at the American Society of Clinical Oncology annual meeting by German Gastric Group. In this study, periop FLOT protocol improved downsizing and complete resection rates and also prognosis in patients with resectable gastric and GEJ cancer compared to periop ECF/ECX [5].

According to the above various factors mentioned, we have adopted the docetaxel-containing triplet neoadjuvant chemotherapy (FLOT) to treat locally advanced gastric cancer recently in our department since 2016. However, most of the results come from Europe [5, 6]. Some researchers thought that FLOT may not be suitable for Asian patients due to the high dose intensity of FLOT and more vulnerable to bone marrow suppression of the Asian population [18, 22]. However, there were few studies exactly revealing the effect and adverse effect of FLOT on Asian patients right now. In our study, patients who received the FLOT protocol showed good tolerability and few side effects. Except for one patient, all the other patients completed four cycles of neoadjuvant chemotherapy. The most side effects were neutropenia, leucopenia, and gastrointestinal toxicity reactions. The previous trial using the FLOT as neoadjuvant chemotherapy resulted in grade 3/4 side effects which were as follows: leucopenia (28%), neutropenia (52%), and anemia (9%) [19, 23]. In our study, grade 3/4 of leucopenia, neutropenia, and anemia occurred in 17.4% followed by 30.4% and 13% of the patients. In the present study, the most occurring side effects were neutropenia and leukopenia, both of which were relieved after symptomatic treatments.

The influence of neoadjuvant chemotherapy on the operative morbidity and mortality is another critical problem due to the degree of toxicity [20, 24]. The most noticeable postoperative complications are surgical site infections including wound infections and intra-abdominal abscess, followed by anastomotic leakage [21, 25]. In the present study, complications following gastrectomy occurred in 6 patients (26%), yet only mild complications were observed. Wound infection (13.0%) was the most observed postoperative complication. Postoperative complications classified as Clavien–Dindo classification grade III or more were only found in one case. It is considered that the FLOT chemotherapy protocol would not increase postoperative complications. FLOT could be acceptable for locally advanced GC.

Tumor size and location [22, 23, 26, 27], histopathology [24, 28], and radical resection (R0) of the primary tumor [25, 26, 29, 30] are found to have a close relationship with the prognosis of resected GC. For locally resectable advanced gastric cancer, the surgeon should make every effort to achieve complete resection of all gross disease to avoid the possibility of microscopic margin positivity [31]. In addition, with neoadjuvant chemotherapy, more advanced stage gastric cancer patients can achieve a higher complete negative microscopic margin of resection, making a positive influence on OS. In our study, the R0 resection rate was 91.3%, which was superior to that achieved in the FLOT4-AIO study (85%) [19]. However, a simple single comparison of the R0 rate with that from the previous FLOT4-AIO trial is not justified, due to many probable differences in patient conditions.

The therapeutic effectiveness of the FLOT protocol was also indicated by the high pathological response rate and pCR rate. Achieving pCR after neoadjuvant chemotherapy is normally associated with a better OS and DFS [27, 32]. The previous retrospective study revealed that pCR can be increased by about 15% using preoperative docetaxel-based triplet neoadjuvant chemotherapy [28, 33]. Recent research shows that the histopathological complete regression with FLOT was higher than other regimens. The pCR percentage was 17.4% in Hamann's study [29, 34] and 16% in Al-Baran's study [19, 23]. Our result showed 13% of the pCR rate.

Laparoscopic gastrectomy has been recommended to treat early gastric cancer [30, 35]. The efficacy of laparoscopic gastrectomy in patients with locally advanced gastric cancer has been only demonstrated in a few studies [31, 36]. Recent evidences advocate laparoscopic surgery for locally advanced GC. Korean KLASS-02 trail was a phase-III multicenter RCT which compared surgical and oncologic safety of laparoscopic distal gastrectomy with open surgery for locally advanced gastric cancer [32, 37]. It indicated that the early morbidity rate and hospital stay were lower in laparoscopic surgery. The result favors laparoscopic distal D2 gastrectomy to treat locally advanced GC. Chinese CLASS-01 trial was a multicenter prospective RCT to evaluate the surgical safety and long-term outcomes of laparoscopic gastrectomy compared with conventional open surgery for advanced GC. The result of postoperative recovery was faster in laparoscopic surgery as KLASS-02. Even more importantly, it showed that postoperative complication rates after laparoscopic gastrectomy for advanced GC were acceptable and comparable to open surgery [33, 38]. It released the result of a 3-year DFS rate (76.5% in laparoscopic gastrectomy versus 77.8% in open distal gastrectomy) recently [7]. These results may support the use of laparoscopic gastrectomy to treat locally advanced cancer. But in the CLASS-01 trial, none received neoadjuvant chemotherapy. Whether laparoscopic surgery after neoadjuvant chemotherapy is suitable for advanced gastric cancer is being disputed. Only two randomized phase II trials were proposed to be carried out in China and Japan separately, but the results have not yet to be published [34, 35, 39, 40]. Based on the existing evidences, we used laparoscopic D2 gastrectomy for locally advanced GC in our hospital. Another reason was that if the patients were not suitable for surgery, the laparoscopy can be served as an exploration method to avoid making a big wound. Our results showed that all 23 patients receiving laparoscopic D2 gastrectomy had enough number of lymph nodes harvested, low blood loss, and fast postoperative recovery. An optimal technique of digestive tract reconstruction after distal gastrectomy has not yet been established. Some studies reported that Roux-en-Y gastrojejunostomy can prevent alkaline reflux gastritis, esophagitis, dumping syndrome, and carcinogenesis of the gastric remnant. In our department, we usually used Roux-en Y reconstruction especially in patients who receive neoadjuvant chemotherapy [41]. Although our study only included the data of laparoscopic surgery, the results were comparable to those reported by another retrospective study in which neoadjuvant chemotherapy was oxaliplatin-containing doublet or triplet regimen (SOX: oxaliplatin and TS-1; CAPOX: oxaliplatin and capecitabine; FOLFOX7: oxaliplatin, leucovorin, and 5-fluorouracil) [36, 42]. And our results show much higher CR and PR compared with the study due to the therapeutic effectiveness of FLOT. We thought that laparoscopic gastrectomy after neoadjuvant chemotherapy can have safety and efficacy as an open gastrectomy. But it still needs a high level of evidences.

There are also some limitations to our study. Its retrospective nature may induce some bias. The short period of follow-up may have impacted our results. In our study, no control treatment group and small study sample size may also reduce the credibility of the result. The result needs to be further verified in prospective, randomized controlled trials in the future.

## 5. Conclusion

Our results suggest that FLOT neoadjuvant chemotherapy, followed by laparoscopic D2 gastrectomy, is effective in advanced, resectable advanced gastric cancer. Preoperative FLOT was tolerable and a good option for patients with resectable gastric cancer. Laparoscopic D2 gastrectomy can be safely performed after such neoadjuvant chemotherapy.

## Figures and Tables

**Table 1:** Patient demographics (*n* = 23).

	Number
Age, median (range)	63 (35–74)

Sex	
Male	18
Female	5

Histological subtype	
Well-differentiated	2
Moderately differentiated	3
Poor differentiated adenocarcinoma	16
Signet-ring	2

Clinical T stage	
T3	9
T4a	14

Clinical N stage	
N1	10
N2	13

Clinical TNM stage	
IIB	5
IIIA	9
IIIB	9

**Table 2:** Adverse events during neoadjuvant chemotherapy (*n* = 23).

Toxicities	Grade 1-2	Grade 3	Grade 4	% grade 3/4
Hematologic			0	
Leukopenia	6	4	0	17.4
Neutropenia	7	7	0	30.4
Thrombocytopenia	0	0	0	0
Anemia	4	3	0	13.0

Gastrointestinal				
Nausea	10	3	1	17.4
Vomiting	5	1	1	8.7
Diarrhea	6	1	0	4.3
Constipation	4	2	0	8.7
Anorexia	3	3	0	13.0

Laboratory				
AST	5	0	0	0
ALT	5	0	0	0

Fatigue	2	0	0	0
Neurosensory	1	0	0	0

AST, aspartate transaminase; ALT, alanine transaminase.

**Table 3:** Operative findings and postoperative outcomes (*n* = 23).

	Number
Peritoneal dissemination	
Negative	23
Positive	0
Residual tumor	
R0	21
R1	2
Type of resection	
Total gastrectomy	12
Distal gastrectomy	11
Type of reconstruction	
Billroth I	8
Roux-en Y	15
Operative time (min)	
Median (range)	255 (195–310)
Resected lymph nodes	
Median (range)	25 (19–38)
Intraoperative bleeding (ml)	
Median (range)	105 (50–350)
The first aerofluxus time (days)	2.7 (1–6)
The first defecating time (days)	5.1 (3–8)
Time to pull drainage (days)	8.5 (5–24)
Time to liquid diets (days)	6.8 (4–9)
Postoperative hospital stays (days)	13.2 (8–31)

**Table 4:** Postoperative morbidity.

Clavien–Dindo classification	Number
Grade I	
Wound	3

Grade II	
Pneumonia	2
Pancreatic fistula	1

Grade IIIa	
Postoperative bleeding	0
Intra-abdominal abscess	1
Anastomotic stenosis	0
Leakage	0

**Table 5:** Pathological findings (*n* = 23).

	Number
Depth of tumor invasion (ypT)	
T0	3
T1	3
T2	7
T3	9
T4a	1

Lymph node metastasis (ypN)	
N0	12
N1	7
N2	4

ypStage	
0	3
I	7
II	10
III	3

Histological response	
TRG1a	3
TRG1b	6
TRG2	8
TRG3	6

## Data Availability

The data used to support the findings of this study are available from the corresponding author upon request.
